# Redundancy of Terms in Search Strategies. Comment on “Searching PubMed to Retrieve Publications on the COVID-19 Pandemic: Comparative Analysis of Search Strings”

**DOI:** 10.2196/28666

**Published:** 2021-05-28

**Authors:** Daniel Melo De Oliveira Campos, Umberto Laino Fulco, Jonas Ivan Nobre Oliveira

**Affiliations:** 1 Universidade Federal do Rio Grande do Norte Natal Brazil

**Keywords:** coronavirus, COVID-19, pandemic, scientific publishing, PubMed, literature searching, research, literature, search, performance, search strategy

Recently, a very interesting study on the performance of different search strategies for COVID-19 records in PubMed was published in the *Journal of Medical Internet Research* [1]. In this article, Lazarus et al compared the performance of PubMed’s one-click search option with both simpler and more complex search strings. Novice and expert searchers do well to keep these in mind when searching. For instance, a search strategy for a review is a time-consuming endeavor, and energy spent on locating relevant controlled vocabulary and keywords can be undermined by errors in formatting, compilation, and translation of these terms. Unfortunately, the presence of these errors is extremely common even among published studies. Sampson and McGowan [2] reviewed studies published in Cochrane and discovered that 90.5% of their sample had a search strategy that contained one or more errors. Some related to errors regarding term, or term variant, identifications, but others pertained to errors in the formatting and basic compilation of the terms. The latter category included Boolean errors (19%), incorrect line numbers (1.6%), the use of Medical Subject Headings (MeSH) and free text terms combined on the same line (20.6%), and the search strategy not being appropriately translated for other databases (20.6%).

In 2018, a study with a random sample of 70 Cochrane Reviews found problems in the design of the search strategies in 73% of reviews, and 53% of these contained problems that could limit both the sensitivity and precision of the search [3]. Recently, Salvador-Oliván et al (2019) [4] evaluated the search strategies of 137 systematic reviews in PubMed to identify errors, analyze their impact on information retrieval, and propose solutions. The results of this study reveal that the percentage of search strategies that contain various types of errors is quite high (92.7%) and that 78.1% of these errors affect recall. Although a substantial proportion of the errors came from inadequate identification of terms, errors were also introduced at the formatting level, with an absence of field tags (21.2%) and lack or incorrect use of quotation marks (5.8%), Boolean operators (1.5%), and parentheses (5.1%) [4].

As to be expected, some errors have graver effects on results than others. Errors that have no effect at all on the number of results include redundant terms and morphological repetition; these “search errors” do not affect recall or negatively affect information retrieval with respect to either recall or precision.

An example of redundancy is as follows: “2019 novel coronavirus disease”[tw] OR “2019 novel coronavirus infection”[tw] OR “2019-nCoV disease”[tw] OR “2019-nCoV infection”[tw] OR “COVID-19 pandemic”[tw] OR “COVID-19 virus disease”[tw] OR “COVID-19 virus infection”[tw] OR “COVID19”[tw] OR “SARS-CoV-2 infection”[tw] OR “coronavirus disease 2019”[tw] OR “coronavirus disease-19”[tw] OR “COVID-19 pandemic”[tw] OR “COVID-19”[tw]. Authors justify redundancy because the decision to include or exclude terms depends on the references retrieved, as the effect of the terms on the results is impossible to predict. However, it is known beforehand that the first 11 terms in a PubMed search can be easily discarded because using the 12th variation will cover all 11, so other terms are unnecessary.

In terms of the search process, tools pertaining to data mining have been developed to help librarians identify relevant terms. Some text-mining approaches have been documented by Stansfield et al [5], including TFIDF, Termine, and BibExcel. Also recommended are librarian tools that often have a particular focus on the MeSH thesaurus, such as PubMed PubReMiner [6] and Yale MeSH Analyzer [7] for keywords and controlled vocabulary.

Created and updated by the United States National Library of Medicine, MeSH vocabulary is used by the ClinicalTrials.gov registry to classify which diseases are studied by the trials registered in its database. This hierarchically organized terminology for indexing and cataloging of biomedical information is divided into four types of terms. The main terms are the “headings” (also known as MeSH headings or descriptors), which describe the subject of each article. Most of these are accompanied by a list of synonyms or very similar terms (known as entry terms). When performing a MEDLINE search via PubMed, entry terms are automatically translated into (ie, mapped to) the corresponding descriptors with a good degree of reliability. In this sense, we highlighted the importance of using the controlled vocabulary “COVID-19” (unique id: C000657245) and “SARS-CoV-2” (unique id: D000086402) in PubMed searches focused on COVID-19–related studies, and not the set of terms (search 1, 2, 3, 6, 7, and 8) analyzed by Lazarus and collaborators [1].

Redundant terms in a search strategy do not affect the retrieval of information; however, the principle of parsimony instructs us to eliminate that which is unnecessary. Applied to information retrieval, this principle prompts us to eliminate any terms or phrases from a search strategy that do not retrieve or provide new records, as they are thus unnecessary.

## Abbreviations

MeSH: Medical Subject Headings
